# Predictive Factor of Local Recurrence after Balloon-Occluded TACE with Miriplatin (MPT) in Hepatocellular Carcinoma

**DOI:** 10.1371/journal.pone.0103009

**Published:** 2014-07-21

**Authors:** Toru Ishikawa, Satoshi Abe, Ryousuke Inoue, Tomoyuki Sugano, Yuhsuke Watanabe, Akito Iwanaga, Keiichi Seki, Terasu Honma, Takeo Nemoto, Keiko Takeda, Toshiaki Yoshida

**Affiliations:** 1 Department of Gastroenterology and Hepatology, Saiseikai Niigata Daini Hospital, Niigata, Japan; 2 Department of Radiology, Saiseikai Niigata Daini Hospital, Niigata, Japan; University of Pisa, Italy

## Abstract

**Background:**

Miriplatin (MPT) is a novel platinum complex used in TACE that shows promise for the treatment of hepatocellular carcinoma (HCC). However, rapid washout has been reported in some cases. Therefore, various methods of administration with MPT have been attempted to increase its therapeutic efficacy. One hopeful method is balloon-occluded TACE (B-TACE), but the therapeutic efficacy of B-TACE with MPT has not been evaluated.

**Aim:**

To investigate the treatment outcomes and factors involved in local recurrence after B-TACE with MPT in HCC.

**Methods:**

This study included 51 patients (55 nodules) with HCC lesions equal or less than 5 cm in diameter who underwent B-TACE with MPT between January 2012 and June 2013. Local recurrence after B-TACE with MPT and factors associated with local recurrence were evaluated.

**Results:**

The overall local recurrence rate was 11.1% at 6 months and 26.2% at 12 months. The local recurrence rate did differ significantly depending on CT values immediately after B-TACE with MPT. Multivariate analysis also showed that the CT value after B-TACE with MPT was the only factor related to local recurrence after B-TACE.

**Conclusions:**

B-TACE with MPT achieves relatively good local control of HCC. The plain CT value immediately after B-TACE with MPT is a predictive factor for local recurrence. In patients with unsatisfactory CT values, locoregional therapy or additional treatment is required.

## Introduction

Hepatocellular carcinoma (HCC) is one of the most common malignant tumors worldwide [1], [2]. Transarterial chemoembolization (TACE) has been established for the treatment of hepatocellular carcinoma (HCC) when surgical resection or other local treatment is not indicated [3], [4]. The major local effects of TACE include local retention of anticancer drugs and lipiodol and the ischemic effects of the embolic material. Various anticancer drugs may be selected, including doxorubicin [5], epirubicin [6]–[8], mitomycin C [9], and cisplatin [10], [11]. However, the use of these drugs varies among medical centers, and the superiority of any particular drug in terms of effectiveness has not been established.

Miriplatin (cis-[((1R,2R)-1,2-cyclohexanediamine-N,N0)bis(myristato)]-platinum(II)monohydrate; Dainippon Sumitomo Pharma Co., Ltd, Osaka, Japan) is a novel lipophilic cisplatin derivative that can be suspended in lipiodol [12]–[15].

It has been developed as a new drug for use in transcatheter arterial infusion (TAI) for HCC [16]. However, the recent study reported that the local recurrence rate was significantly higher for miriplatin compared to epirubicin with mitomycin C in the lipiodol based superselective TACE for HCC [17]. This inferior local control with MPT can be attributed to its higher viscosity.

Various methods of administration with MPT are currently being attempted to increase the therapeutic efficacy of MPT. Irie et al. [18] revealed that dense accumulation in the HCC nodule could be achieved by B-TACE with doxorubicin and mitomycin C.

We speculated that B-TACE with MPT may be dense accumulation in the HCC to overcome inferior local control.

The aim of this study was to evaluate treatment outcomes and factors associated with local recurrence in patients with HCC lesions equal or less than 5 cm who underwent B-TACE with MPT.

## Patients and Methods

### 1. Patients

A total of all 51 patients (55 nodules) with HCC who underwent consecutively recruited unto the study protocol of B-TACE using miriplatin (Miripla; Dainippon Sumitomo Pharma, Co., Ltd., Osaka, Japan) from January 2012 to June 2013 at Saiseikai Niigata Daini Hospital. The study protocol was approved and written informed consent was obtained from all participating patients.

Before treatment of B-TACE using miriplatin, all patients underwent a comprehensive evaluation consisting of a measurement of tumor size, liver-imaging studies (dynamic computed tomography [CT], ultrasonography [US], digital-substraction angiography [DSA]), complete blood count and blood chemistry. Diagnosis of HCC was established based on the findings of dynamic CT, US and DSA.

The study exclusion criteria were: 1) tumor size ≥5 cm; 2) intentionally incomplete TACE because of tumor infiltration; 3) ≥4-month interval between TACE and initial CT during follow-up observation; 4) no CT done during follow-up observation; and 5) nodules with locoregional therapy such as percutaneous ethanol injection (PEI), microwave coagulation therapy, laser ablation, and radiofrequency (RF) ablation as additional treatment, 6) extrahepatic metastasis of HCC, 7) other malignancies.

### 2. Methods

In all patients, the femoral artery was punctured using the Seldinger technique, a 5-Fr introducer was inserted, and then a 5-Fr hook shaped catheter (MS-kit; Medikit, Tokyo, Japan) was inserted into the celiac artery. Then, a 3-Fr microcatheter balloon catheter (Attendant, Teromo, Tokyo, Japan) was advanced using a coaxial technique into the feeding arteries of each tumor. The microballoon catheter was introduced over a 0.014-inch guide wire (Chikai; Asahi Intec, Aichi, Japan). The location of microballoon catheter tip was aimed artery. The balloon was inflated to a diameter 5–10% larger than that of the occluded artery. MPT infusion was performed after balloon occlusion. MPT infusion was continued under balloon occlusion until HCC was filled with MPT or portal venous branches were beginning to be filled with MPT. Fluoroscopy and DSA during B-TACE procedures observed whether limitation of MPT inflow into nontumourous liver parenchyma and dense accumulation in HCC nodule were present, and whether or not anastomotic vessels with collateral artery were present. Then balloon was deflated.

For B-TACE using MPT, each 70-mg vial of MPT was dissolved in 4 mL of lipiodol to prepare, at most, an 8-mL suspension of lipiodol with 140 mg of MPT. MPT was injected into the occluded artery under the inflated balloon catheter until the HCC nodule was filled with MPT.

Plain CT scans were performed immediately after B-TACE with MPT as post B-TACE examination before heading back to ward. This CT value of lipiodol retained in the HCC nodule was measured by using a region of interest (ROI) in the tumor. ROI dimensions were identical for each patient. The lesion density means in Hounsfold units (HU) on CT.

All CT images were obtained using a multidetector-row helical CT scanner with a GE Light -Speed VCT scanner (GE Japan, Tokyo, Japan) with a 3-mm collimation, 5-mm-thick sections, and 5-mm reconstruction intervals.

Multidetector CT images were evaluated using Image J 1.38 software (NIH, Betheda, MD, USA) to measure the attenuation of the target lesions in Hounsfield Units (HU).

During follow-up observation after treatment, local recurrence and lipiodol retention were evaluated by subsequent CT scans. All patients were followed up every 3 months with measurement of serum alpha-fetoprotein (AFP), and des-gamma-carboxy prothrombin (DCP), and enhanced CT or enhanced MRI. Detection of local tumor progression was defined a recurrent tumor within or adjacent to the treated tumor.

Local recurrence was defined as either of the following: 1) an area without lipiodol retention within the tumor after treatment, with enhancement in the contrast early phase, and hypodensity in the late phase; or 2) an area adjacent to lipiodol retention with enhancement in the contrast early phase, and hypodensity in the late phase. Any lesion separated from lipiodol retention, even if only slightly, was not regarded as local recurrence.

The severity of adverse reactions was evaluated during the first treatment cycle according to the Common Terminology Criteria for Adverse Events v4.0 (CTCAE v4.0). All patients gave their informed consent after being fully informed on this study, and were enrolled in this study specifically and followed prospectively. and. The study was approved by the Ethical Committee of Saiseikai Niigata Daini hospital and was conducted in accordance with the principles of the Declaration of Helsinki.

### 3. Statistical Analysis

Prior to this study, all demographic and clinicopathological data had been prospectively collected in a computer database. Specifically, the following variables parameters were used for analyzing the risk factors for recurrence: age, sex, etiology, serum tumor markers [alpha-fetoprotein (AFP) and des-gamma-carboxy prothrombin (DCP)], total bilirubin, serum albumin, prothrombin activity, Child-Pugh score, size of tumors and CT retention value after B-TACE with MPT. CT retention value was measured after B-TACE immediately. Recurrence rates were estimated using the Kaplan–Meier method and differences between groups were compared using the log-rank test. Multivariate analysis to identify independent prognostic factors was carried out using the Cox proportional hazards model to calculate the adjusted hazard ratio (HR) and 95% confidence interval (CI). Statistical processing was performed using StatView version 5.0 software (SAS Institute, Cary., N.C., USA). All reported P values are 2-sided, with P<0.05 considered statistically significant.

## Results

### Patients Background

Table 1 summarizes the clinical background characteristics of all patients. There were 35 men and 16 women, with a mean age of 70.90±9.17 years. Among the 51 enrolled patients, 10 patients were seropositive for hepatitis B surface antigen, 28 patients were seropositive for hepatitis C virus antibody, and 13 patients were seronegative for both hepatitis B surface antigen and hepatitis C virus antibody.

**Table 1:** Clinical background of 51 patients.

Demographic variables	Mean ± SD	Range
Age (years)	70.90±9.17	47–86
Sex (Male:Female)	35:16	
Etiology (HBV/HCV/NonBNonC)	10/28/13	
AFP (ng/mL)	233.66±583.46	1.3–2801.0
DCP (mAU/mL)	181.55±335.09	12.0–2142.0
Total Bilirubin (mg/dL)	0.65±0.27	0.20–1.45
Serum Albumin (g/dL)	3.64±0.44	2.70–4.50
Prothrombin activity (%)	91.12±13.57	57.90–130.50
Child-Pugh score	6.37±1.34	5.0–11.0
CT value (Hounsfield Unit)	373.55±201.51	63.0–167.37

SD; Standard Deviation, AFP; alpha-fetoprotein, DCP; des-gamma-carboxy prothrombin.

Mean laboratory values were: AFP 233.66±583.46 ng/mL, DCP 181.55±335.09 mAU/mL, T-Bil 0.65±0.27 mg/dL, albumin 3.64±0.44 g/dL, and prothrombin activity 91.12%±13.57%. The mean Child-Pugh score was 6.37±1.34. The mean plain CT value immediately after B-TACE with MPT was 373.55±201.51 HU. Median values: were: AFP 67.20 ng/mL, DCP 168.00 mAU/mL, T-Bil 0.65 mg/dL, albumin 3.60 g/dL, and prothrombin activity 90.30%. The mean Child-Pugh score was 6.0. The mean plain CT value immediately after B-TACE with MPT was 325.7 HU.

### Recurrence Rate

**Figure 1:**
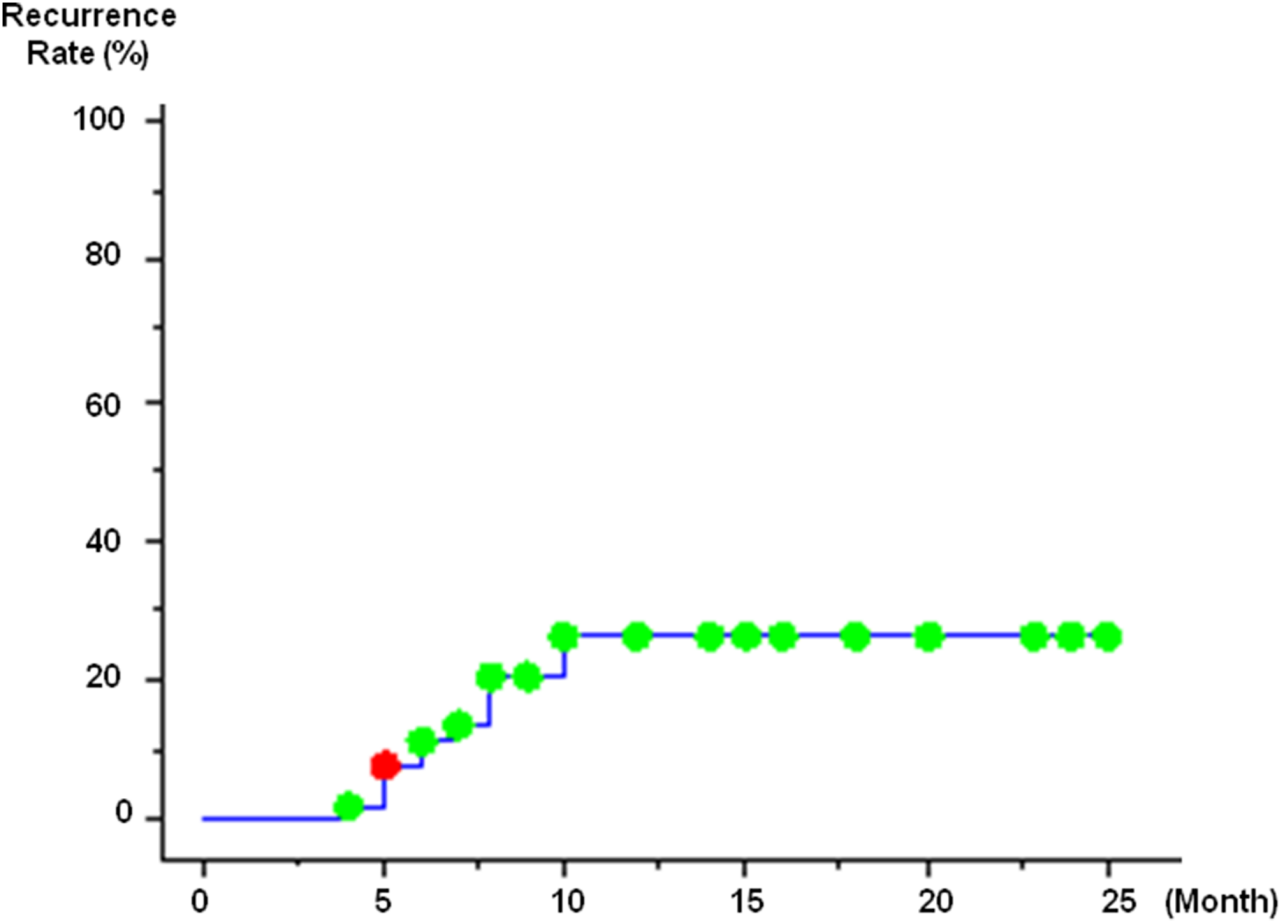
Local recurrences after balloon occuluded transcatheter arterial chemoembolization with miriplatin. The local recurrence rates at 6 months, and 1 year were 11.1, and 26.2%, respectively.

**Figure 2:**
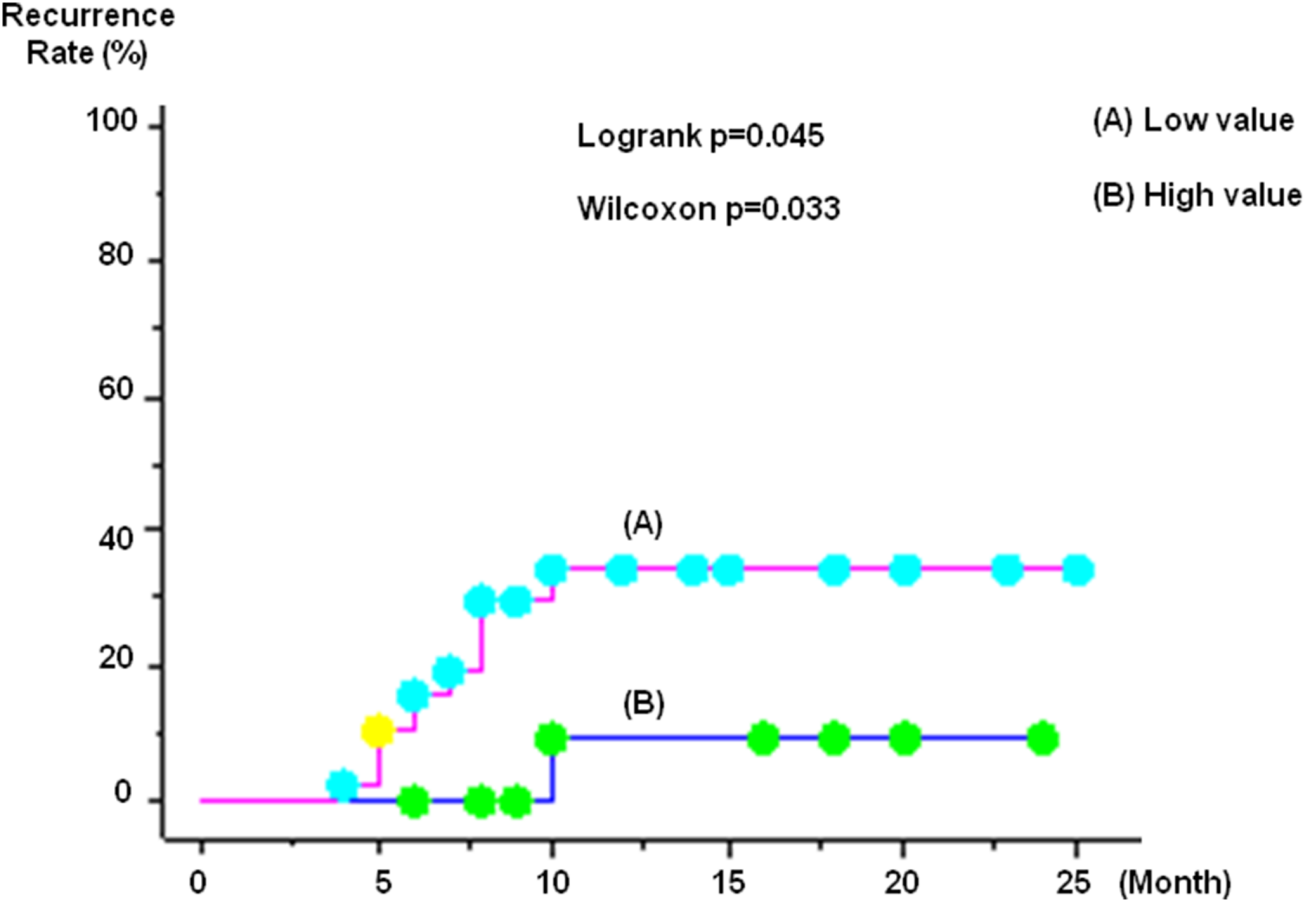
In regard to plain CT results immediately after B-TACE treatment, the local recurrence rate in the higher-than-mean CT value group was 4.8% at 6 months and 16.0% at 12 months, but in the lower-than-mean CT value group, the local recurrence rates were significantly different: 15.2% at 6 months and 32.9% at 12 months.

Among the 55 HCC nodules, there was local recurrence in 12 nodules. The overall local recurrence rate was 11.1% at 6 months and 26.2% at 12 months (Figure 1). Median recurrence time is 9 months. In regard to plain CT results immediately after B-TACE treatment, the local recurrence rate in the higher-than-mean CT value group was 4.8% at 6 months and 16.0% at 12 months, but in the lower-than-mean CT value group, the local recurrence rates were significantly different: 15.2% at 6 months and 32.9% at 12 months (Figure 2).

### Factors related to local recurrence

No significant differences in local recurrence rates were observed due to age, sex, etiology, serum tumor markers [alpha-fetoprotein (AFP) and des-gamma-carboxy prothrombin (DCP)], total bilirubin, serum albumin, prothrombin activity, Child-Pugh score, size of tumors. CT value immediately after B-TACE with MPT was correlated with local recurrence.

Multivariate analysis showed that only the CT value immediately after B-TACE with MPT (hazard ratio 0.11; 95% confidence interval 0.01–0.98; p = 0.048) was the significantly correlated with local recurrence (Table 2).

**Table 2:** Risk factors for local recurrence after MPT-B-TACE.

Variables	Univariate		Multivariate	
	HR(95% CI)	P-value	HR(95% CI)	P-value
Gender (male)	0.43(0.09–2.01)	0.294		
Age (>65 years)	0.70(0.15–3.34)	0.649		
Size (<30 mm)	0.97(0.16–5.83)	0.968		
AFP (>100 ng/mL)	1.25(0.20–7.92)	0.816		
DCP (>100 mAU/mL)	0.24(0.05–1.16)	0.076		
Child-Pugh (A)	0.67(0.19–2.41)	0.541		
CT value (High)	0.11(0.01–0.98)	0.048	0.12(0.01–0.97)	0.049

HR; Hazard Ratio, CI; Confidence Interval, AFP; alpha-fetoprotein, DCP; des-gamma-carboxy prothrombin.

#### Adverse effect

Fever (78.4%), anorexia (31.3%), and elevation of serum transaminase levels were observed in most patients after B-TACE. The following Grade3 or Grade 4 adverse events were observed: increased AST in 19 patients (37.3%), increased ALT in 8 patients (15.7%); all these cases resolved within 2 weeks. In this study group, no vascular complications of the hepatic artery were observed. No other serious complications or treatment-related deaths were observed after B-TACE.

## Discussion

Anticancer drugs used with TACE for treatment of hepatocellular carcinoma (HCC) have included doxorubicin [5], epirubicin [6]–[8], mitomycin C [9] and cisplatin [10], [11], either as monotherapy or as combination therapy. The recent introduction of platinum agents has widened the options for anticancer drug treatment. Various anti-cancer drugs as above have been used as TACE agents for the treatment of HCC. However, the most effective and least toxic TACE protocol for HCC has yet to be identified. Miriplatin (Miripla; Dainippon Sumitomo Pharma, Osaka, Japan) is a third generation platinum derivative and has been developed recently for transarterial treatment of HCC [13]–[15]. In Japan, the “Guideline on the Use of New Anticancer Drugs for the Treatment of Hepatocellular Carcinoma” was prepared by the Study Group on New Liver Cancer Therapies established by the “Research Project on Emergency Measures to Overcome Hepatitis” under the auspices of the Health and Labour Sciences Research Grant. In this study, miriplatin is facilitate regarding the proper usage of new anticancer drugs towards actual use in therapy [19].

Following intra-arterial administration, the miriplatin-iodized oil suspension accumulates in the target tumor, and continuous antitumor effects caused by gradual release of active platinum compounds are expected [13]–[15]. In general, miriplatin is suspended in an oily contrast medium and injected through the hepatic artery without successive embolization with porous gelatin particles. It has been reported, however, that the tumor necrosis is more extensive in cases receiving TACE than in those receiving transarterial infusion chemotherapy, and that the former procedure yields superior survival rates as compared with the latter [20], [21].

This novel feature of miriplatin can be potentially beneficial for long-acting antitumor effects, thus making it a superior chemotherapeutic agent as compared to other hydrophilic agents. In addition, since the oil-suspended miriplatin remains in the tumor for a long period, its rapid release into the systemic circulation is inhibited, resulting in reduced systemic side effects such as nausea/vomiting, renal damage, and other acute toxic events [16].

Miriplatin is as a new drug less vascular disorders for use in transcatheter arterial infusion (TAI) for HCC. However, Miyayama et al. [17] reported that superselective TACE using MPT resulted in very frequent local recurrence despite less arterial damage rather than TACE using epirubicin and mitomycin C.

It is reported that MPT/Lipiodol suspension cannot enter small vessels due to high viscosity. This may lead to insufficient delivery to distal tumor vessels, early washout, and lack of an antitumor effect. Therefore, methods of administration with MPT have been devised to enable delivery to distant tumor vessels.

We argue that this is a potentially important improvement, as previous studies using miriplatin for TACE have reported higher local recurrence rates due to rapid drug washout.

One hopeful method for improving treatment is using B-TACE. Irie et al. [18] reported that they have used a 3F microballoon catheter in selective transarterial chemoembolization treatment for HCC to prevent proximal migration and leakage of embolization materials. They have noticed dense lipiodol emulsion (LE) accumulation in HCC nodule by selective balloon-occluded transarterial chemoembolization (B-TACE). Because dense LE accumulation in HCC increases accumulation of anticancer drugs, it is important to reveal the mechanism for this dense LE accumulation.

With advances in catheters, B-TACE was developed using microcatheters to further improve the local control effects of standard TACE. B-TACE is a treatment method not simply for embolization under occlusion, but rather, by altering hemodynamics under balloon occlusion, more effective accumulation of lipiodol emulsion is achieved, thus improving local control. Furthermore, Irie et al. reported that, by using B-TACE to limit flow of lipiodol into normal parenchyma, inflow of the lipiodol emulsion into HCC nodules continued, thus achieving more dense accumulation.

MPT Study of no B-TACE does not provide informed consent ethically as above data. Therefore, MPT treatment of our institution has become B-TACE one arm all. B-TACE with MPT in the present study, relatively good outcomes were achieved, with local recurrence rates of only 11.1% at 6 months and 26.2% at 12 months. This data is similar to superselective TACE using epirubicin and mitomycin C, and superior to superselective MPT TACE by Miyayama et.al [17].

We speculate MPT stasis in the peripheral vessels supplying the liver parenchyma and MPT continued to flow into the HCC nodules with dense accumulation by balloon occlusion. Furthermore, this MPT stasis by balloon occlusion may change viscosity of MPT.

Plain CT immediately after MPT B-TACE macroscopically suggested satisfactory lipiodol accumulation, but there was variation in these plain CT values. Taking into consideration, these differences in accumulation are likely due to the presence of communicating arteries [22], with flow of MPT outside of the tumor.

In the present study, no significant differences in local recurrence rates were observed due to age, sex, etiology, serum tumor markers [alpha-fetoprotein (AFP) and des-gamma-carboxy prothrombin (DCP)], total bilirubin, serum albumin, prpthrombin activity, Child-Pugh score, size of tumors. The local recurrence rate did differ significantly depending on whether CT values just after B-TACE were higher than the mean value. The results of the present study showed that plain CT values immediately after MPT B-TACE are important in predicting local recurrence.

Although B-TACE is useful to effectively utilize MPT as an anticancer drug, satisfactory CT values must be achieved to further reduce local recurrence. If these are unsatisfactory, locoregional therapy such as RFA, PEIT and MCT or additional TACE may be necessary to achieve additive effects with MPT. Further prospective studies of treatment strategy in a larger number of patients are required.
